# Diet Quality and History of Gestational Diabetes Mellitus Among Childbearing Women, United States, 2007–2010

**DOI:** 10.5888/pcd12.140360

**Published:** 2015-02-26

**Authors:** Rui S. Xiao, Tiffany A. Moore Simas, Sharina D. Person, Robert J. Goldberg, Molly E. Waring

**Affiliations:** Author Affiliations: Tiffany A. Moore Simas, University of Massachusetts Medical School and University of Massachusetts Memorial Health Care, Worcester, Massachusetts; Sharina D. Person, Robert J. Goldberg, Molly E. Waring, University of Massachusetts Medical School, Worcester, Massachusetts.

## Abstract

**Introduction:**

Women with a history of gestational diabetes mellitus (GDM) have elevated risk of developing type 2 diabetes. Diet quality plays an important role in the prevention of type 2 diabetes. We compared diet quality among childbearing women with a history of GDM with the diet quality of childbearing women without a history of GDM.

**Methods:**

We used data from the National Health and Nutrition Examination Survey for 2007 through 2010. We included women without diabetes aged 20 to 44 years whose most recent live infant was born within the previous 10 years and who completed two 24-hour dietary recalls. The Healthy Eating Index (HEI)-2010 estimated overall and component diet quality. Multivariable linear regression models estimated the association between a history of GDM and current diet quality, adjusting for age, education, smoking status, and health risk for diabetes.

**Results:**

A history of GDM was reported by 7.7% of women. Compared with women without a history of GDM, women with a history of GDM had, on average, 3.4 points lower overall diet quality (95% confidence interval [CI], −6.6 to −0.2) and 0.9 points lower score for consumption of green vegetables and beans (95% CI, −1.4 to −0.4). Other dietary component scores did not differ by history of GDM.

**Conclusion:**

In the United States, women with a history of GDM have lower diet quality compared with women who bore a child and do not have a history of GDM. Improving diet quality may be a strategy for preventing type 2 diabetes among childbearing women.

## Introduction

Gestational diabetes mellitus (GDM), defined as glucose intolerance that is first detected during pregnancy, affects 2% to 10% of pregnant women in the United States each year (1). Although serum glucose levels typically return to prepregnancy levels shortly after delivery, women with GDM have a 35% to 60% chance of developing type 2 diabetes over the next 10 to 20 years, 7 times the risk of women without GDM (2). A diagnosis of GDM provides an opportunity to intervene to prevent type 2 diabetes following pregnancy for this at-risk population.

Evidence has increasingly demonstrated that lifestyle interventions and behavior modification have the potential to delay or prevent progression to type 2 diabetes in high-risk populations such as adults with prediabetes (3,4) and women with a history of GDM (3,5). Studies show that a good-quality overall diet is better for preventing type 2 diabetes than a diet enriched by a single nutrient or dietary component (6,7). Diet quality measures how closely people’s diets align with national dietary guidelines and how diverse the healthy choices are within core food groups (8). Poor diet quality is associated with significantly higher risk of chronic diseases (6,9,10) and premature death (11).

Adherence to a healthy diet is associated with a lower risk of type 2 diabetes among women with a history of GDM (12). Many postpartum women with a history of GDM have poor diet quality (13), including fruit and vegetable intake being less than the amount recommended to prevent type 2 diabetes (14,15). However, there is little evidence regarding the overall diet quality of women with and women without a history of GDM. The objective of this study was to examine diet quality in relation to a history of GDM in a nationally representative sample of childbearing women in the United States. We hypothesized that women with a history of GDM would have poorer diet quality than women without a history of GDM.

## Methods

The National Health and Nutrition Examination Survey (NHANES) is a cross-sectional survey of a nationally representative sample of the noninstitutionalized American population. NHANES employs a complex multistage probability cluster design. Full details of the sampling methodology and data collection procedures used in this study have previously been published (16). In brief, participants were interviewed by trained, mostly bilingual, interviewers for demographic, socioeconomic, dietary, and health-related information at their homes and at a mobile examination center. Beginning in 1999, NHANES became a continuous program with data collection every 2 years. Because information about a history of GDM was first collected in 2007 through 2008, we combined NHANES cycles 2007 through 2008 and 2009 through 2010 for this analysis to increase our available cohort size. Our cohort consisted of women without diabetes aged 20 to 44 years whose most recent live-born infant was delivered within the previous 10 years. The University of Massachusetts Medical School Institutional Review Board deemed this study exempt from human subjects research oversight.

Women reported their history of GDM as part of the reproductive health questionnaire. They were asked, “During any of your pregnancies, were you ever told by a doctor or other health professional that you had diabetes, sugar diabetes, or gestational diabetes?” The response options included yes, no, borderline, refused, and I don’t know. Women who replied yes to this question were classified as having a history of GDM. Women selecting the options borderline, refused, or I don’t know were excluded from the analysis.

During their examination in the mobile examination center, women completed a 24-hour dietary recall with trained interviewers. They completed a second 24-hour dietary recall via telephone 3 to 10 days later. During both interviews, women listed detailed information on the foods and beverages they consumed from midnight to midnight on the day before the interview. To describe the women’s typical diet, we averaged their food intake reported during these 2 recalls and used this average food intake to assess their diet quality. We calculated the Healthy Eating Index (HEI)-2010 score to measure diet quality according to the recommendations of the Dietary Guidelines for Americans (17). The HEI-2010 is a valid and reliable measure of diet quality (18). The higher the HEI-2010 scores, the better the women’s compliance with dietary guidelines and the better the overall diet quality. The HEI-2010 has 12 components, resulting in a maximum total score of 100 points. Six components (total fruit, whole fruit, total vegetables, greens and beans, total protein foods, and seafood and plant proteins) contribute 0 to 5 points each; 5 components (whole grains, dairy, fatty acids, refined grains, and sodium) contribute 0 to 10 points each; and 1 component (empty calories from solid fats, alcohol, and added sugars) contributes 0 to 20 points. We used documented SAS code (SAS Institute, Inc) (19), MyPyramid Equivalents Database (MPED), Center for Nutrition Policy and Promotion (CNPP) MyPyramid Equivalents Databases, and CNPP Addendum to the MyPyramid Equivalents Database (MPED, version 2.0B) to calculate total and component HEI-2010 scores. For refined grains, sodium, and empty calories from solid fats, alcohol, and added sugars, lower intakes receive more points. For the other 9 components, higher intakes receive higher points. We summed the 12 component scores to estimate a total HEI-2010 diet quality score, which ranged from 0 to 100.

We considered age, race/ethnicity, education level, poverty-to-income ratio, marital status, weight, physical activity level, smoking status, diagnosis of prediabetes, and having health risks for diabetes as potential confounders because of observed associations of these factors with diet quality. With the exception of weight, these data were self-reported. Race/ethnicity was categorized as non-Hispanic white, non-Hispanic black, Mexican-American/Hispanic, and other race/ethnicity (including multiracial). Education level was categorized as less than high school graduate, high school graduate or general equivalency diploma, some college or an associate’s degree, and college graduate or higher. Household poverty-to-income ratio, calculated by NHANES, is the ratio of household income to the poverty threshold for a household of that size and is categorized as less than 1, 1 to less than 3, and 3 or more. Body mass index (BMI) was calculated from height and weight (kg/m^2^), as measured by trained staff, and was categorized as underweight (BMI <18.5), normal weight (BMI 18.5–24.9), overweight (BMI 25.0–29.9), and obese (BMI ≥30) (20). Participants were asked a series of questions about the frequency and duration of various physical activities over the previous 30 days or in a typical week. Women were categorized as achieving or not achieving 30 minutes of moderate physical activity on 5 or more days a week, 20 minutes of vigorous physical activity on 3 or more days a week, or an equivalent combination, as recommended for US adults (21). Smoking status was determined by asking participants, “Have you smoked at least 100 cigarettes in your entire life?” Persons who replied no were classified as never smokers. Persons who replied yes were asked, “Do you now smoke cigarettes?” Those who replied that they smoked every day or on some days were classified as current smokers. Those who replied no were classified as former smokers. Participants who reported that a doctor or other health professional had told them that they had prediabetes, impaired fasting glucose, impaired glucose tolerance, or borderline diabetes, or that their blood glucose was higher than normal but not high enough to be called diabetes or sugar diabetes were considered to have prediabetes. Participants who reported that they had ever been told by a doctor or other health professional that their health conditions or medical or family history increased their risks for diabetes were considered to have health risk for diabetes.

All statistical analyses were weighted. Sample weights account for the complex sampling scheme of NHANES and for oversampling of certain population subgroups and survey nonresponse. Weighted analyses produce estimates that are representative of the US civilian noninstitutionalized population (16). As recommended in the NHANES analytic guidelines (16), we created a combined 4-year dietary weight by assigning one-half of the 2-year dietary weight for each survey cycle (2007–2008 or 2009–2010).

Characteristics of the study women in relation to a history of GDM were compared by using χ^2^ tests for categorical variables and *t* tests for continuous variables. Because our analyses estimated the characteristics of childbearing women nationally, we provided standard errors (SEs) for mean values and 95% confidential intervals for proportions to indicate the variability in these estimated values. The primary study outcome was overall diet quality, as assessed by the total HEI-2010 score. We used linear regression models to estimate the association between a history of GDM and total HEI-2010 score. Potential confounders were added into the model if they had bivariate associations with the HEI-2010 total score with *P* < .25 and were retained in the adjusted model if their inclusion changed the regression coefficient for diet quality by 10% or more. Secondarily, we constructed multivariable-adjusted linear regression models to estimate the association between a history of GDM and each HEI-2010 component score, adjusting for these covariates. All analyses were conducted using SAS version 9.3.

## Results

A total of 2,557 women aged 20 to 44 years completed the reproductive history questionnaire. We excluded women who had never delivered a live-born infant (n = 1,013), those who were currently pregnant (n = 125), and those who had their last live-born infant more than 10 years previously (n = 434) or did not report when their most recent live infant was born (n = 23). We further excluded women who reported a physician’s diagnosis of diabetes (n = 22) or borderline diabetes treated by oral hypoglycemics (n = 2), women reporting previous borderline GDM (n = 17), and those who did not complete 2 dietary recalls (n = 168; 18 did neither recall, 147 completed only 1 recall, and 3 had a second recall that was deemed unreliable by interviewers), resulting in an analytic cohort of 817 women.

GDM during a previous pregnancy was reported by 7.7% (95% confidence interval [CI], 5.6–9.9) of US women. Women who had a history of GDM were, on average, 2.5 years older than women without such a history (34.6 vs 32.1; Table 1). Women who had a history of GDM were less likely to be of non-Hispanic white race/ethnicity (51.4% vs 60.5%) and were more likely to be obese (54.5% vs 32.9%), to report a diagnosis of prediabetes (7.4% vs 2.0%), and to report health risks for diabetes (24.3% vs 12.2%) (Table 1).

**Table 1:** Characteristics of Study Women^a^ in Relation to History of Gestational Diabetes Mellitus (GDM), National Health and Nutrition Examination Survey (NHANES), 2007–2010

Characteristic	History of GDM	No History of GDM	*P* Value
**Sample N**	77	740	NA
**Weighted N**	1,478,744	17,646,633	
**Age, y, mean (SE)**	34.6 (0.8)	32.1 (0.4)	<.01
**Race/ethnicity, weighted % (95% CI)**			
Non-Hispanic white	51.4 (34.0–68.8)	60.5 (51.4–69.6)	.38
Non-Hispanic black	14.5 (4.4–24.7)	13.0 (8.8–17.2)	
Mexican-American/Hispanic	25.2 (12.5–37.9)	22.6 (16.0–29.2)	
Other race/ethnicity	8.9 (0.0–18.7)	3.9 (1.5–6.3)	
**BMI status,^b^ kg/m^2^, weighted % (95% CI)**			
Underweight (<18.5)	1.5 (0.0–4.6)	2.0 (0.3–3.7)	.01
Normal weight (18.5–24.9)	18.5 (8.7–28.2)	35.8 (29.9–41.8)	
Overweight (25.0–29.9)	25.5 (13.4–37.6)	29.3 (23.0–35.7)	
Obese (≥30)	54.5 (39.2–69.8)	32.9 (29.0–36.7)	
**Parity, weighted % (95% CI)**			
1	13.0 (1.0–24.9)	28.0 (22.9–33.4)	.10
2	38.6 (21.1–56.2)	40.2 (33.8–46.5)	
3	32.1 (17.9–46.3)	23.3 (19.0–27.7)	
≥4	16.3 (6.0–26.7)	8.5 (5.8–11.2)	
**Education level, weighted % (95% CI)**			
Less than high school graduate	30.7 (17.1–44.2)	20.7 (16.8–24.6)	.40
High school graduate or GED	22.4 (8.6–36.2)	20.7 (16.0–25.5)	
Some college or associate’s degree	30.9 (15.6–46.3)	33.2 (28.7–37.8)	
College graduate or above	16.0 (2.2–29.7)	25.3 (20.0–30.7)	
**Marital status,^b^ weighted % (95% CI)**			
Married or living with partner	78.6 (67.9–89.2)	76.5 (72.6–80.4)	.52
Widowed, divorced, or separated	12.6 (3.1–22.1)	9.8 (6.8–12.7)	
Never married/single	8.8 (1.7–16.0)	13.7 (10.7–16.8)	
**Poverty-to-income ratio,^b^ weighted % (95% CI)**			
<1	30.8 (18.9–42.8)	25.4 (22.0–28.7)	.74
≥1 but <3	36.9 (20.9–52.9)	39.4 (34.4–44.4)	
≥3	32.2 (14.1–50.3)	35.2 (29.2–41.3)	
**Smoking status, weighted % (95% CI)**			
Never smoker	58.1 (42.2–74.0)	61.7 (56.0–67.5)	.90
Former smoker	14.6 (0.02–29.1)	13.1 (9.9–16.4)	
Current smoker	27.3 (15.7–38.9)	25.1 (19.3–30.9)	
**Met recreational physical activity guidelines, weighted % (95% CI)**	30.3 (16.6–43.9)	31.7 (25.5–38.0)	.85
**Prediabetes,^b^ weighted % (95% CI)**	7.4 (1.0–13.9)	2.0 (0.6–3.5)	.02
**Health risks for diabetes,^b^ weighted % (95% CI)**	24.3 (10.9–37.7)	12.2 (9.5–14.9)	.02

Abbreviations: BMI, body mass index; CI, confidence interval; GED, general equivalency diploma; SE, standard error.

a Women aged 20 to 44 years who gave birth to at least 1 live infant in the 10 years before the NHANES survey.

b Missing data for BMI (n = 1), marital status (n = 1), poverty-to-income ratio (n = 54), prediabetes (n = 3), and having health risks for diabetes (n = 1).

For all women in the cohort, the overall average diet quality score was 49.3 out of 100 (SE, 1.1; range: 14.7–90.9). Average diet quality score was 46.4 (SE, 1.6; range: 23.0–76.4) among women with a history of GDM and 49.5 (SE, 1.1; range: 14.7–90.9) among women without a history of GDM. After we adjusted for age, education, smoking status, and diabetes risk, women with a history of GDM had, on average, 3.4 points lower total diet quality compared with women without a history of GDM (95% CI, −6.4 to −0.2) (Table 2). The only component diet quality score that differed by history of GDM was the consumption of greens and beans; women with a history of GDM had average scores 0.9 points lower for consumption of these foods than women without a history of GDM (95% CI, −1.4 to −0.4; mean [SE], 1.0 [0.2] vs 1.7 [0.1], respectively) (Table 2).

**Table 2:** Diet Quality in Relation to History of Gestational Diabetes (GDM) Among Study Women^a^ in the United States, National Health and Nutrition Examination Survey (NHANES), 2007–2010

Diet Element	Maximum HEI-2010 Score	History of GDM, Mean (SE)	No History of GDM, Mean (SE)	Crude β (95% CI)	Multivariable-Adjusted^b^ β (95% CI)
Total diet quality	100	46.4 (1.6)	49.5 (1.1)	−3.1 (−6.5 to 0.3)	−3.4 (−6.6 to −0.2)
Total fruit	5	2.3 (0.3)	2.4 (0.2)	−0.1 (−0.7 to 0.5)	−0.1 (−0.7 to 0.5)
Whole fruit	5	2.1 (0.3)	2.4 (0.1)	−0.2 (−0.9 to 0.4)	−0.3 (−1.0 to 0.4)
Total vegetable	5	3.0 (0.2)	3.3 (0.1)	−0.3 (−0.7 to 0.2)	−0.3 (−0.9 to 0.2)
Greens and beans	5	1.0 (0.2)	1.7 (0.1)	−0.7 (−1.2 to −0.2)	−0.9 (−1.4 to −0.4)
Total protein foods	5	4.5 (0.1)	4.3 (0.04)	0.2 (−0.1 to 0.4)	0.2 (−0.1 to 0.4)
Seafood and plant protein	5	2.5 (0.3)	2.3 (0.1)	0.2 (−0.5 to 0.8)	0.1 (−0.5 to 0.8)
Whole grain	10	1.7 (0.3)	2.2 (0.1)	−0.5 (−1.0 to 0.02)	−0.4 (−1.0 to 0.2)
Dairy	10	4.7 (0.4)	5.8 (0.2)	−1.1 (−2.1 to −0.1)	−0.9 (−2.0 to 0.2)
Fatty acids	10	4.5 (0.6)	4.6 (0.2)	−0.1 (−1.2 to 1.1)	−0.02 (−1.1 to 1.1)
Sodium	10	4.7 (0.5)	4.2 (0.2)	0.5 (−0.6 to 1.6)	0.3 (−0.8 to 1.5)
Refined grain	10	5.3 (0.5)	5.4 (0.2)	−0.04 (−1.1 to 1.1)	0.0 (−1.1 to 1.1)
Empty calories	20	10.1 (1.0)	11.1 (0.5)	−0.9 (−2.9 to 1.1)	−1.0 (−2.7 to 0.7)

Abbreviations: CI, confidence interval; HEI, Healthy Eating Index.

a Women aged 20 to 44 years who gave birth to at least 1 live infant in the 10 years before the NHANES survey.

b Adjusted for age, education, smoking status, and diabetes risk.

## Discussion

We found that US women who bore at least 1 live infant during the previous 10 years had, on average, poor diet quality and that overall diet quality was worse among women with a history of GDM. Women with a history of GDM have a markedly elevated risk for developing type 2 diabetes compared with women without GDM (2). To prevent type 2 diabetes, the American College of Obstetrics and Gynecology (22) and American Diabetes Association (23) recommend that all women at increased risk for the disease be counseled about the benefits of a healthy and balanced diet, exercise, and weight management. Our findings highlight the need for public health and clinical attention on diet quality among childbearing women, particularly those with a history of GDM.

Women reported suboptimal consumption of most diet-quality components, with mean scores close to 50% of the maximum score. Average consumption of greens and beans and whole grains was particularly low, with scores approximately 15% to 20% of the maximum. Women with a history of GDM had significantly lower consumption of greens and beans compared with women without a history of GDM. These foods may be of particular benefit for reducing type 2 diabetes risk, and their consumption should be encouraged among childbearing women, particularly those with a history of GDM. One recent systematic review and meta-analysis showed that greater consumption of green leafy vegetables was associated with a 14% reduction in the risk of type 2 diabetes (24). Beans and peas are great sources of protein, fiber, and many vitamins and minerals. One review suggested that replacing high energy-dense food with beans and peas would have beneficial effects on the prevention and management of obesity and other comorbidities (25). Consumption of beans could also improve overall diet quality (26). Women with a history of GDM should be counseled to include more greens and beans to improve their diet quality and meet dietary recommendations to reduce the risk of type 2 diabetes.

In addition to links with increased chronic disease risk for women (6,9,10), maternal diet quality is a significant contributor to children’s diet quality (27), and women who modify their diet typically make comparable changes to their children’s diet (28). In a study of women with children at home, women with a history of GDM were less likely to meet the national guidelines for fruit and vegetable consumption (14). Improving diet quality among childbearing women, especially those with a history of GDM, has the potential for positive intergenerational health effects.

Our study has several strengths and limitations. The use of data from a large national health study enabled us to generalize our results to the 19 million noninstitutionalized US women aged 20 to 44 years who gave birth to at least 1 live infant within the previous 10 years. Two 24-hour interviewer-administered dietary recalls produce a better estimate of women’s usual dietary intake than a single recall (29). We assessed diet quality by using the HEI-2010, which is a valid and reliable measure of diet quality in NHANES (18). We used individual food and nutrient data to generate the HEI-2010 diet quality score, which allowed for detailed analysis of overall and individual components of diet quality. Although misclassification is possible, a self-reported history of GDM has a high level of agreement with GDM data gathered from birth certificates (30). Women self-reported their age only at first GDM diagnosis; therefore, we have no knowledge of whether they also had GDM during their most recent pregnancies. NHANES is a cross-sectional survey, and thus, we did not have information on diet quality before pregnancy, during pregnancy, or any time after their most recent pregnancies.

Childbearing women in the United States have, on average, poor diet quality. Women with a history of GDM have significantly lower overall diet quality and reported lower consumption of greens and beans than women without a history of GDM. Given the role of diet quality in the prevention of type 2 diabetes and other chronic diseases, our findings highlight the need for, and importance of, public health and individual clinical interventions to increase consumption of total protein, greens and beans, and whole grains to improve the overall diet quality of women of childbearing age, particularly women with a history of GDM.
